# Antibiogram and beta-lactamase genes among cefotaxime resistant *E. coli* from wastewater treatment plant

**DOI:** 10.1186/s13756-020-0702-4

**Published:** 2020-03-12

**Authors:** Anthony Ayodeji Adegoke, Chibuzor Ezinne Madu, Olayinka Ayobami Aiyegoro, Thor Axel Stenström, Anthony Ifeanyi Okoh

**Affiliations:** 1 SARChI, Institute for Water and Wastewater Technology, Durban University of Technology, Durban, 4000 South Africa; 2 Department of Microbiology, Faculty of Science, University of Uyo, PMB 1018, Uyo, Akwa Ibom State Nigeria; 3 Applied and Environmental Microbiology Research Group (AEMREG), Department of Biochemistry and Microbiology, University of Fort Hare, Alice, South Africa; 4 GI Microbiology and Biotechnology Unit, Agricultural Research Council- Animal Production, Irene, 0062 South Africa; 5 SAMRC Microbial Water Quality Monitoring Centre, University of Fort Hare, Alice, South Africa

**Keywords:** Cefotaxime, Carbapenem, Beta-lactamase genes, Wastewater, *bla*_*CTX*_, *bla*_*OXA-1*_, Ciprofloxacin

## Abstract

**Background:**

The World Health Organization (WHO) recently classified Enterobacteriaceae resistance to third-generation cephalosporin into the group of pathogens with critical criteria for future research.

**Methods:**

A study to assess the antibiogram and beta-lactamase genes among the cefotaxime resistant *E. coli* (CREc) from a South African wastewater treatment plant (WWTP) was conducted using standard phenotypic and molecular biology characterization methods.

**Results:**

Approximate total *E. coli* (TEc) concentration (log_10_ CFU/mL) ranged between 5.7 and 6.8 among which cefotaxime resistant *E. coli* were between 1.8 and 4.8 (log_10_ CFU/mL) for cefotaxime antibiotic concentration of 4 and 8 mg/L in the influent samples. Effluent samples, heavily influenced by the chlorination had only 0.3 log_10_ CFU/mL of TEc. Fifty-one cefotaxime resistant isolates were selected out of an overall of 75 isolates, and subjected to a new round of testing, with a follow up of 36 and 48 isolates for both colistin and gentamicin, respectively as guided by initial results. Selected CREc exhibited resistance to amoxicillin-clavulanic acid (35.3%; *n* = 51), colistin sulphate (76.5%; *n* = 36), ciprofloxacin (47.1%; n = 51), gentamicin (87.5%; *n* = 48) and intermediate-resistance to meropenem (11.8%; n = 51). Extended spectrum-beta-lactamase genes detected, viz.: *bla*_*CTX*-M_ (52.6%; *n* = 38) and *bla*_*TEM*_ (84.2%; n = 38) and concurrent *bla*_*CTX-M*_ *+ bla*_*TEM*_ (36.8%; n = 38), but no *bla*_*SHV*_ was detected. Carbapenem resistance genes, *blaKPC*-2 (15.8%; n = 38), *blaOXA*-1 (57.9%; n = 38), *blaNDM*-1 (15.8%; n = 38) were also detected. Approximately, 10.5 - 36.8% (n = 38) co-occurrence of two or more beta-lactamase genes was detected in some isolates. Out of the selected number (*n* = 30), 7(23.3%) were enterotoxigenic *E. coli* (ETEC), 14 (46.7%) were Enteroaggregative *E. coli* (EAEC), but no enteropathogenic *E. coli* (EPEC) was detected.

**Conclusion:**

Resistance to cefotaxime and the presence of a wide range of beta-lactamase genes exposed the potential risks associated with these pathogens via occupational and domestic exposure during the reuse of treated wastewater.

## Introduction

*Escherichia coli* has been accepted as both a biological indicator of water contamination and a human pathogen implicated in both systemic (e.g. septicemia, urinary tract infection, meningitis, etc) and superficial infections (skin or wound infection) [1–6]. *E. coli* is part of the normal flora of the gastrointestinal tract, shed along with faecal waste also including release by infected and convalescent individuals through poorly managed wastewater [7, 8]. The public health impact of the *E. coli* released depends partly on the antibiotic susceptibility pattern of the organism, as this determines the clinical treatment option(s) available [9]. In specific terms, Park [10] inferred that resistance to third-generation cephalosporin and carbapenem by Gram-negative bacteria in the community is very difficult to manage. In South Africa, cefotaxime is extensively in use [11–13], as supported by Section 21 of South African constitution [11]. Koopmans et al. [12] reported that cefotaxime is one of the two most used antibiotics in pediatric ward and pediatric intensive care unit of South African hospitals.

On the global level, the resistance of *E. coli* (and other members of the Enterobacteriaceae) to cefotaxime has been categorized to be of a critical threat to the public health by the World Health Organization, WHO [14], for which research is seriously required. The threat associated with the resistance to these groups of antibiotics is due to their use as last line of defense against infections. Bacterial species showing resistance to them usually possess both genes and enzymes capable of conferring resistance to other antibiotics [15, 16].

Resistance to cefotaxime (cephalosporin) is often due to production of enzymes like extended-spectrum beta-lactamases, ESBLs encoded by the beta-lactamase genes, such as *bla*_*CTX-M*_ [16–19]. The same bacterial species containing the *bla*_*CTX-M*_ may also contain *bla*_*TEM*_ and/or *bla*_*SHV*_, especially among clinical isolates [20, 21]. If found in municipal wastewater, they might be an indication of clinical origin [21] and also important, because wastewater treatment plants (WWTPs) are hotspots for antibiotic-resistant bacteria or resistance genes and it can be used as early warning signs on the health of the human population. Diwan et al. [22] showed the detection of 88% *bla*_*CTX-M*_ gene bearing *Enterobacteriaceae* isolates while Korzeniewska and Harnisz [18] reported that 55% of the same types of bacterial strain harbored the gene*.* D’Andrea et al. [19] reported *bla*_*CTX-M*_ as the primary basis of resistance to the third-generation cephalosporins in *E. coli* isolated from clinical and environmental samples. In Nicaragua, *bla*_*CTX*-M_ genes were detected among ESBL-producing *E. coli* from hospital wastewater samples [17].

For cefotaxime resistant *E. coli* with these ESBLs genes, their threat becomes more critical if they also possess carbapenem resistance genes, *bla*_*VIM*_*, bla*_*OXA-1*_*, bla*_*KPC-2*_*, bla*_*NDM-1*_, etc. [23]. Carbapenem resistance genes code for resistance against imipenem, meropenem, ertapenem, doripenem etc. *E. coli* with carbapenem resistance and carbapenem resistance genes in the treated hospital effluent possesses potential epidemiological threat globally, as the bacteria may initiate difficult to treat infection among the exposed groups [24–27]. Transfer of antibiotic resistance genes can also be interspecifically and intraspecifically, given a suitable environment [27]. Most beta-lactamase genes are plasmid based and can easily be transferred in biofilm formed within wastewater systems or in lakes [28, 29]. Exchange of plasmids and other mobile genetic elements containing beta-lactamase genes does occur in water, which serves as cushioning matrix in the process [30]. *E. coli* and other bacteria originating from different sources interact within the wastewater matrix and may modify bacterial ecosystems therein [31], depending on the duration of the interaction. Though WWTPs are to reduce the biological contamination of water, the efficiency of treatment would determine the potential release of bacteria with a critical epidemiological threat like cefotaxime resistant *E. coli*.

Some reports inferred that the conditions in WWTPs give room for the interaction of antibiotic-resistant bacteria and the exchange of mobile genetic elements [30–32]. Surveillance of the release of these WWTPs is imperative, to determine the biological status like the presence of cefotaxime resistant *E. coli* in the effluent that could constitute a threat. The aim of this study, therefore, was to assess and enumerate the cefotaxime resistant *E. coli* in influent and effluent samples of a wastewater treatment plant in Durban, South Africa and the corresponding sensitivity pattern to other selected antibiotics, as well as the presence of ESBL genes and other selected beta-lactamase genes in them.

## Materials and methods

### Description of the sampling sites and sample collection

The sampling was done in a WWTP, treating municipal wastewater from Durban, Kwazulu Natal Province, South Africa in February (summer) and August (winter), 2017. The WWTP receives inflow in the range of 12,000–14,000 m^3^ containing industrial, municipal and hospital inflow. The total influent Chemical Oxygen Demand (COD) was 250–1100 mg/L and the total effluent COD was 75 mg/L. Other WWTP information are tabulated in Table 1. The treatment plant contained four primary settling tanks, six trickling filters, six settling tanks and three anaerobic digesters (unheated and unmixed). The plant was designed to provide service for 30,000 people (design capacity = 18.80ML/d; working capacity = 10.98 ML/d mixed). It contained a chlorination step for water disinfection before release to the recipient.

**Table 1:** Wastewater parameters from the WWTP

Wastewater parameters	Influent (mg/L)	Effluent (mg/L)
COD (total)	250–1100	75
Total-N	10–60	0–25
NH_4_-N	10–60	0–25
Total-P	20–80	0–10
NO_3_-N (Effluent)	N/A	0–10
Suspended solids (Effluent)	N/A	25 μS/cm

N/A means Not Available

In five sampling rounds representing different seasons both pre-grated influent and final (post-chlorinated) effluent samples were collected. The rationale for collecting pre-grated samples was to have the samples in the exact forms it entered the WWTP. To have the samples in the form in which the effluents released to the recipient water, post-chlorinated final effluent samples were collected. The samples transported in a cold chain analyzed within 12 h of collection.

### Quantification and isolation of total *E. coli*, cefotaxime resistant *E. coli*, other coliforms and cefotaxime resistant coliforms

Quantification of the bacteria was done using standard membrane filtration. Membrane Fecal Coliform (mFC) agar (500 g; Difco ref. 267,720) purchased from Quantum Biotech, South Africa was used as the medium for quantification. The media was prepared with 1% rosolic acid (Difco; ref. 232,281) purchased from Hach International. Ten-fold serial dilutions representative for the influent and effluent ranges of concentration were prepared using sterilized saline solution (0.85% (w/v) NaCl). The prepared samples were filtered through 0.2 μm cellulose acetate filters (Lasec, South Africa) in triplicates. The filters were placed on the mFC agar plates supplemented with 4 mg/L or 8 mg/L cefotaxime antibiotic (100 mg Cefotaxime sodium salt; Sigma; ref. C7039) and on agar plates without antibiotic supplements in line with UNESCO regulations for another ongoing project in our laboratory. Though 4 mg/L was optimized to be adequate for satisfactory results, continuation of both 4 mg/L and 8 mg/L throughout was to see potential variance over a while.

The resulting culture was incubated at 37 °C for 24 h. Enumeration of the total number of colonies depended on observation of specific colonial colorations (grey, pink and blue). *E. coli* forms blue colonies. Grey and/or pink colonies might not be coliforms. The selectivity of the media was determined by estimating the ratio of blue and total colonies.

### Species-specific identification of *E. coli*

#### Extraction of DNA

Isolation of the DNA was done by the boiling method using the protocols of Salehi et al. [33]. The young presumptive *E. coli* colonies of ≤24 h were suspended in 200 μL aliquots of distilled water. The suspension containing the isolates was mixed by vortexing until a mixture with thoroughly dissipated isolates formed. This was boiled at 95 °C for 15 min and subsequently centrifuged at 15000 rpm; at 4 °C for 15 min where after, the supernatant was removed and stored at − 20 °C for further assays.

#### PCR based species-specific identification of *E. coli*

PCR based species-specific identification of *E. coli* was done using the primers (Inqaba Biotechnical Industries, South Africa) specific for a conserved region of *E. coli* alanine racemase (*alr*) gene specified in Table 2 [34, 35]. The reaction mixture contained 12.5 μL of one taq quick load 2x master mix with standard buffer (Inqaba Biotechnical Industries, South Africa), 20 μM each of the forward and reverse primers, water and 3 μL of DNA template suspension in a final volume of 25 μL.

**Table 2:** Primer sequences and amplicon size of PCR-amplified gene targets

Target species	Gene target	Primer sequence 5′–3′	Amplicon size (bp)
*E. coli*	*Alr*	CTGGAAGAGGCTAGCCTGGACGAG	366
		AAAATCGGCACCGGTGGAGCGATC	

The cycling conditions for the PCR reaction started with an initial 6 min denaturation step at 95 °C, followed by 35 cycles containing denaturation at 95 °C for 20s, primer annealing/extension at 72 °C for 1 min 30 s, and a final extension for 5 min at 72 °C. *E. coli* WG5 was used as a positive control.

Agarose gel electrophoresis was performed at 80 V for 45 min in 2% agarose gel in Tris Acetate-EDTA (TAE) buffer, stained with Sybr Safe DNA gel stain (ThermoFisher Scientific, South Africa).

### Antibiotic susceptibility testing (AST) in cefotaxime-resistant *E. coli*

Phenotypic antibiotic susceptibility pattern of the selected and identified cefotaxime-resistant *E. coli* was performed following standards described by Cheesebrough [36] and Clinical and Laboratory Standards Institute (CLSI) (CLSI M100–2017) [37]. Fifty-one (51) out of 75 selected isolates were subjected to first-round antibiogram. This was with the selection of 36 and 48 isolates for both colistin and gentamicin, respectively, based on the results from the first series of antibiotics tested. The antibiotics used included meropenem (10 μg), colistin (10 μg), amoxicillin-clavulanic (30 μg), ciprofloxacin (5 μg), trimethoprim-sulphamethoxazole (1.25/23.75 μg), gentamicin (10 μg), tetracycline (30 μg) and nitrofurantoin (300 μg).

#### Multiple antibiotic resistance (MAR) index of cefotaxime-resistant *E. coli* to other antibiotics

Multiple antibiotic resistance index (MAR Index) was determined per isolate as the ratio of the number of antibiotics to which an isolate showed resistance (X) to the total number of antibiotics against which the isolate was tested (Y) [38–40].

$$ \mathrm{MARI}=\mathrm{X}/\mathrm{Y} $$

This was determined on all the isolates concerning all the antibiotics used.

### Detection of genes for extended spectrum beta-lactamases and carbapenemases in cefotaxime-resistant *E. coli*

#### Multiplex PCR for bla_CTX_, bla_TEM_ and bla_SHV_

Multiplex PCR assay was carried out to detect *bla*_*CTX*_*, bla*_*TEM*_ and *bla*_*SHV*_ (Table 3). The PCR reaction mixture of 25 μL contained 12.5 μL of one taq quick load 2x master mix with standard buffer (Inqaba Biotechnical Industries, South Africa), 20 μM each of the forward and reverse primers, water and 3 μL of DNA template suspension. The cycling conditions for the PCR assay included an initial denaturation at 95 °C for 5 min, 30 cycles at 95 °C for 30 s and 68 °C for 40 s and a final extension at 68 °C for 3 min.

**Table 3:** Primer sequences, genes for beta-lactamase and the expected amplicon sizes

Group	Gene name	Primer sequence (5′–3′)	Expected amplicon size (bp)
ESBLS genes	***bla***_***TEM***_	GTCGCCGCATACACTATTCTCA	258
		CGCTCGTCGTTTGGTATGG	
	***bla***_***CTX*-*M***_	CGGGAGGCAGACTGGGTGT	381
		TCGGCTCGGTACGGTCGA	
	***bla***_***SHV***_	GCCTTGACCGCTGGGAAAC	319
		GGCGTATCCCGCAGATAAAT	
Carbapenemase genes	***bla***_***VIM***_	GATGGTGTTTGGTCGCATA	390
		CGAATGCGCAGC	
	***bla***_***OXA-1***_	TTCTGTTGTTTGGGTTTCGC	190
		ACGCAGGAATTGAATTTGTTC	
	***bla***_***KPC*-*2***_	GCTTCCCACTGTGCAGCTCATTC	213
		CGCCCAACTCCTCAGCAACAATTG	
	***bla***_***NDM-1***_	GGTGCATGCCCGGTGAAATC	660
		ATGCTGGCCTTGGGGAACG	

Agarose gel electrophoresis also performed in 2% agarose gel in Tris Acetate-EDTA (TAE) buffer, stained with Sybr Safe DNA gel stain (ThermoFisher Scientific, South Africa).

#### Singleplex PCR for the detection of *bla*_*VIM*_*, bla*_*OXA-1*_*, bla*_*KPC-2*_*, bla*_*NDM-1*_

Singleplex PCR was performed for the detection of *bla*_*VIM*_*, bla*_*OXA-1*_*, bla*_*KPC-2*_*, bla*_*NDM-1*_ using the primers listed in Table 3. For *bla*_*VIM*_*,* the cycling conditions included the initial denaturation at 94 °C for 3 min, followed by a stage of 36 cycles containing denaturation at 94 °C for 1 min, annealing at 55 °C for 1 min, extension at 72 °C for 1 min and a final extension at 72 °C for 5 min. PCR cycling conditions for *bla*_*OXA-1*_ began with initial denaturation at 95 °C for 2 min, followed by a stage of 30 cycles containing denaturation at 94 °C for 45 s, annealing at 55 °C for 30 s and extension at 72 °C at 1 min. This was followed by a final extension stage at 72 °C for 5 min.

The detection of *bla*_*KPC-2*_ was done with cycling conditions that included an initial denaturation at 95 °C for 5 min, followed by a stage of 40 cycles containing denaturation at 95 °C for 15 s, annealing at 66.1 °C for 30 S, extension at 72 °C for the 30 S and final extension at 72 °C for 10 min [41]. For *bla*_*NDM-1*_, the cycling conditions began with denaturation at 95 °C for 15 min, followed by a stage of 30 cycles containing 95 °C for 1 min denaturation, 61.1 °C for 1 min annealing, and extension for the 30 S at 72 °C. This was followed by a final extension for 10 min at 72 °C [42].

#### Pathotyping of the *E. coli*

Isolated and identified *E. coli* were further pathotyped to determine which strains they were, using the primers listed in Table 4. For molecular characterization of *E. coli* pathotypes, the thermal cycling conditions for Enteroaggregative *E. coli* (EAEC), Enteropathogenic *E. coli* (EPEC) and Enterotoxigenic *E. coli* (ETEC) (Heat Labile, LT) were as follows: the initial denaturation/enzyme activation step at 95 °C for 15 min, followed by 35 cycle consisting of denaturing at 94 °C for 45 s, annealing at 55 °C for 45 s, extension at 68 °C for 2 min and the final elongation at 72 °C for 5 min [43]. The cycling conditions for EIEC were: 96 °C for 4 min; 35 cycle of 94 °C for 30 s, 58 °C for 30 s and 72 °C for 1 min; and a final 7 min extension at 72 °C [44] while those for ETEC (Heat Stable, ST) were as follows: 95 °C for 5 min, followed by 30 cycles of 95 °C for 1 min, 60 °C for 1 min, 72 °C for 1 min, and 72 °C for 10 min final extension [45].

**Table 4:** Primer sequences and expected amplicon sizes of the pathogenic Strains of *E. coli* (Pathotyping)

Target strain	Gene Target	Primer Sequence (5′–3′)	Amplicon Size (bp)
EAEC	*Eagg*	AGA CTC TGG CGA AAG ACT GTA TC	194
		ATG GCT GTC TGT AAT AGA TGA GAA C	
EPEC	*EaeA*	CTG AAC GGC GAT TAC GCG AA	917
		GAC GAT ACG ATC CAG	
ETEC	*LT*	GGC GAC AGA TTA TAC CGT GC	450
		CGG TCT CTA TAT TCC CTG TT	
	*ST*	ATTTTTCTTTCTGTATTGTCTT	190
		CACCCGGTACAAGCAGGATT	
EIEC	*shig*	CTGGTAGGTATGGTGAGG	320
		CCAGGCCAACAATTATTTCC	

### Statistical analysis

Statistical analysis was performed for descriptive statistics. Plating of the samples was done in triplicates. The values were plotted using Box and Whiskers, Microsoft Office Excel 2016 (Microsoft, Redmond, WA, USA) which automatically calculated and depicted the standard deviations (SDs) and the spread of data. Differences in antibiotic resistance between ESBL positive *E. coli* and carbapenemase positive *E. coli* were determined using the chi-square test at *p* value < 0.05 (significant). The test of significance for seasonal variation (*p* < 0.05) was done using GraphPad software, version 5.01.

## Results

### Quantification of total *E. coli* and cefotaxime-resistant *E. coli*

**Fig. 1:**
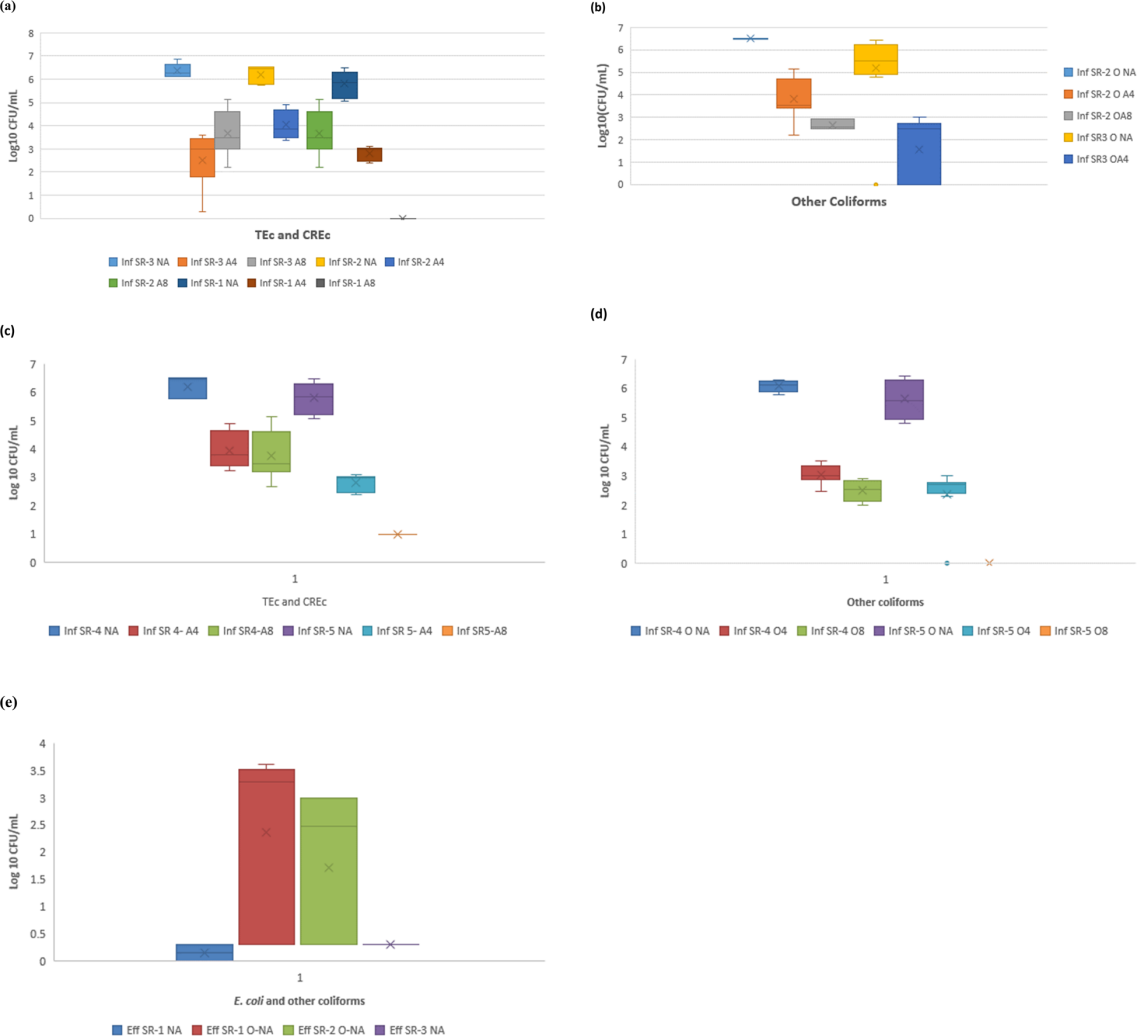
**a** Count [Log_10_ (CFU/mL)] of Presumptive Total *E. coli* (TEc) and Cefotaxime Resistant *E. coli* (CREc) in summer Influent samples (**b**) Count [Log_10_ (CFU/mL)] of other Coliforms and Cefotaxime Resistant Coliforms in summer influent samples. Legend: Inf SR-3 NA = influent sample at sampling occasion 3 without antibiotic, Inf SR-3 A4 = influent sample at sample occasion 3 with 4 mg/L of cefotaxime antibiotic, Inf SR-3 A8 = influent sample 3 with 8 mg/L of cefotaxime antibiotic; Inf SR-2 NA = influent sample at sampling occasion 2 without antibiotic; Inf SR-2 A4 = influent sample at sample occasion 2 with 4 mg/L of cefotaxime antibiotic; Inf SR-2 A8 = influent sample 2 with 8 mg/L of cefotaxime antibiotic; Inf SR-1 NA = influent sample at sampling occasion 1 without antibiotic; Inf SR-1 A4 = influent sample at sample occasion 1 with 4 mg/L of cefotaxime antibiotic; Inf SR-1 A8 = influent sample 1 with 8 mg/L of cefotaxime antibiotic. Figure 1: **c** Count [Log_10_ (CFU/mL)] of Presumptive Total *E. coli* (TEc) and Cefotaxime Resistant *E. coli* (CREc) in Winter Influent samples (**d**) Count [Log_10_ (CFU/mL)] of other Coliforms and Cefotaxime Resistant Coliforms in Winter Influent samples. **e** Count [Log_10_ (CFU/mL)] of *E. coli* and other coliforms in effluent samples. Legend: Inf SR-4 NA = influent sample at sampling occasion 4 without antibiotic, Inf SR-4 A4 = influent sample at sample occasion 4 with 4 mg/L of cefotaxime antibiotic; Inf SR-4A8 = influent sample during sample occasion 4 with 8 mg/L of cefotaxime antibiotic; Inf SR-4 NA = influent sample at sampling occasion 4 without antibiotic; Inf SR-5 A4 = influent sample at sample occasion 5 with 4 mg/L of cefotaxime antibiotic; Inf SR-5 A8 = influent sample during sample occasion 5 with 8 mg/L of cefotaxime antibiotic; Eff SR-1 NA = effluent sample occasion 1 for *E. coli* without antibiotic supplementation; Eff SR-2 NA = effluent sample occasion 2 for *E. coli* without antibiotic supplementation; Eff SR-3 NA = effluent sample occasion 3 for *E. coli* without antibiotic supplementation; Eff SR-1 O-NA = effluent sample occasion 1 for other coliforms without antibiotic supplementation etc.

Large counts of cefotaxime-resistant *E. coli* were resident in the wastewater. A very high mean total *E. coli* count of over 6 log_10_ CFU/mL was found in the influent sample (represented with Inf SR-3 NA in Fig. 1). Varying concentrations of cefotaxime-resistant *E. coli* occurred with differences between the two concentrations of the antibiotic supplemented in the laboratory cultivation media. Higher numbers of resistant isolates were enumerated at cefotaxime concentration of 4 mg/L than at 8 mg/L as assumed, though this was inconsistent at one sampling occasion. Higher concentration of cefotaxime (8 mg/L) did not show reduction in the cefotaxime resistant *E. coli* counts beyond what lower concentration showed in a slight variation.

An approximate total *E. coli* count (log_10_ CFU/mL) ranged from 5.7 to 6.8, with cefotaxime resistant *E. coli* count (log_10_ CFU/mL) ranging between 1.8 to 4.9 for cefotaxime concentration of 4 mg/L and 2.3 to 2.8 at 8 mg/L. The influent counts (log_10_ CFU/mL) for the total *E. coli* count (NA) as well as cefotaxime-resistant *E. coli* (A4 and A8) for all the summer samples (SR-1, SR-2, SR-3) are presented in Fig. 1(a). The results of the other coliforms for summer as depicted in Fig. 1(b) contained a total count (log_10_ CFU/mL) that ranged from about 6.4 to 6.6, with one wide outlier of count 0, plotted separately from the range 6.4–6.6. The cefotaxime (4 mg/L) resistant coliforms counts (log_10_ CFU/mL) ranged from 0 to 4.7 while those for 8 mg/L ranged from about 2.6 to 2.9, presented in Fig. 1(b).

Seasonal variation was not significant (*p* < 0.05) with an approximate total *E. coli* count (log_10_ CFU/mL) in winter that ranged from 5.2 to 6.6, cefotaxime-resistant *E. coli* count (log_10_ CFU/mL) ranged from 2.5 to 4.8 at concentration of 4 mg/L and 1.0 to 5.2 at concentration of 8 mg/L. The trend was also similar for other coliforms with a total count (log_10_ CFU/mL) that ranged from about 4.7 to 6.3; cefotaxime (4 mg/L) resistant coliforms counts (log_10_ CFU/mL) ranged from 2.3 to 3.5 and cefotaxime (8 mg/L) resistant coliforms counts (log_10_ CFU/mL) ranged from 0 to 2.9. The winter counts of *E. coli* and the other coliforms are represented in Fig. 1(c) and (d), respectively.

Meanwhile, there was consistently no total and cefotaxime-resistant *E. coli* growth and rarely very low growth (range in log_10_ CFU/mL = 0 to 0.3) in the effluent concentration (Fig. 1e). Therefore, the reduction efficiencies for total *E. coli* were in the range from 95.6 to 100%, while those estimated for cefotaxime-resistant were between 99 to 100%. Total coliforms in the effluent samples (without cefotaxime) however showed some appreciable counts (log_10_ CFU/mL) that ranged from 0 to 3.6 as in Fig. 1(e). Their removal efficiencies inconsistently ranged from 45.5 to 100%. The majority of the coliforms were sensitive to cefotaxime and did not grow on cefotaxime plates. There were more effluent samples without *E. coli* and coliform growth. Almost all the cefotaxime-supplemented plates for effluent showed no growth. The count of 1 CFU/mL in very few cases was estimated as 0 log_10._

### Antibiogram of cefotaxime-resistant *E. coli*

The results of the antibiotic susceptibility testing from cefotaxime resistant *E. coli* to additional antibiotics is presented in Table 5 together with percentage intermediates and susceptible isolates. Cefotaxime resistant *E. coli* also showed 35.3% resistance to amoxicillin-clavulanic acid, 76.5% to colistin sulphate, 47.1% to ciprofloxacin, and 87.5% to Gentamicin.

**Table 5:** Susceptibility profile of cefotaxime resistant *E. coli* to other selected antibiotics

Antibiotics	Percentage of isolates		
	R	I	S
Amoxicillin-clavulanic acid	35.3	52.9	11.8
^a^Colistin sulphate	76.5	11.8	5.9
Meropenem	0	11.8	88.2
Ciprofloxacin	47.1	23.5	29.4
Trimethoprim-sulphamethoxazole	81.3	0	18.8
^b^Gentamicin	87.5	12.5	0
Tetracycline	56.3	0	37.5
Nitrofurantoin	37.5	25	37.5

*R* Resistance, *I* Intermediate, *S* Sensitive, *n* = 51; ^a^*n* = 36; ^b^*n* = 48

### Multiple antibiotic resistance index (MAR index)

**Fig. 2:**
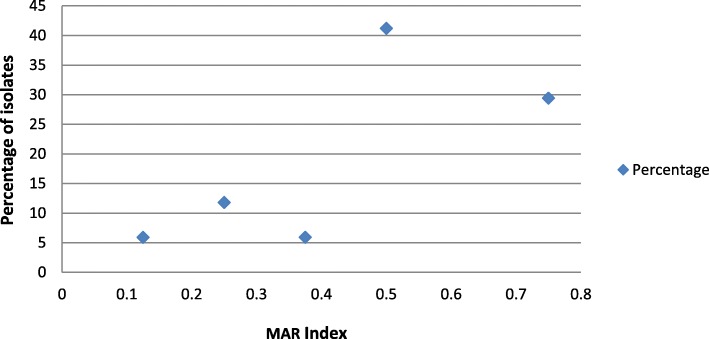
MAR Index of the cefotaxime resistant *E. coli* and their percentage of isolates

A large percentage (> 94%) of the *E. coli* isolates had a MAR index above 0.2. The corresponding figures were 41.2% with a MAR index of 0.5 and 29.4% with an index of 0.75 (See Fig. 2).

### Bata lactamase genes in cefotaxime-resistant *E. coli*

The presence of various beta-lactamase genes among the cefotaxime-resistant *E. coli* occurred*.* For extended-spectrum beta-lactamase (ESBL) genes, *bla*_*CTX-M*_ was detected in 52.6% of the isolates and *bla*_*TEM*_ in 84.2%, while no *bla*_*SHV*_ was detected. Both *bla*_*CTX-M*_ and *bla*_*TEM*_ were concurrently detected among 36.8% of the *E. coli* isolate. Carbapenem resistance gene, *bla*_*KPC-2*_ was detected among 15.8% of the isolates, *bla*_*OXA-1*_ among 57.9% of the *E. coli* isolates, while *bla*_*NDM − 1*_ occurred in 15.8% of the *E. coli* isolates. No *bla*_*VIM*_ was detected. Carbapenem-resistance genes, *bla*_*KPC-2*_ and *bla*_*OXA-1*_ were found in 15.8% of the isolates. *bla*_*NDM-1*_ and *bla*_*OXA-1*_ were also concurrently detected in 10.5%. Both ESBLs genes and carbapenemase genes were concurrently detected in some isolates, with the highest being *bla*_*TEM*_ and *bla*_*OXA-1*_ simultaneously detected in 52.6% of the isolates. Fewer isolates showed the presence of the putative genes for carbapenemases than ESBLs. In addition, there was statistically significant difference (*p* < 0.05) between most carbapenem-resistance genes (*bla*_*KPC-2,*_*bla*_*NDM-1,*_*bla*_*KPC-2 +*_*bla*_*OXA-1*_ and *bla*_*NDM-1 +*_*bla*_*OXA-1)*_ and ESBL genes (*bla*_*CTX-M*_ and *bla*_*TEM*_). The details of the beta-lactamase genes are displayed in Table 6.

**Table 6:** Selected beta-lactamase genes in cefotaxime resistant *E. coli* from wastewater treatment plants

Group	Beta-lactamase genes	%
ESBLs genes (A) *n* = 38	*blaCTX-M*	52.6
	*blaTEM*	84.2
	*blaSHV*	0
	*blaCTX-M + blaTEM*	36.8
Carbapenem genes (B) *n* = 38	*blaKPC-2*	15.8
	*blaOXA-1*	57.9
	*blaVIM*	0
	*blaNDM-1*	15.8
	*blaKPC-2 + blaOXA-1*	15.8
	*blaNDM-1 + blaOXA-1*	10.5
A + B *n* = 38	*blaCTX-M + blaKPC*	10.5
	*blaCTX-M* + *blaOXA-1*	36.8
	*blaCTX-M* + *blaNDM-1*	10.5
	*blaTEM* + *blaKPC-2*	15.8
	*blaTEM* + *blaOXA-1*	52.6
	*blaTEM* + *blaNDM-1*	15.8

### Pathotypes of the isolates

**Fig. 3:**
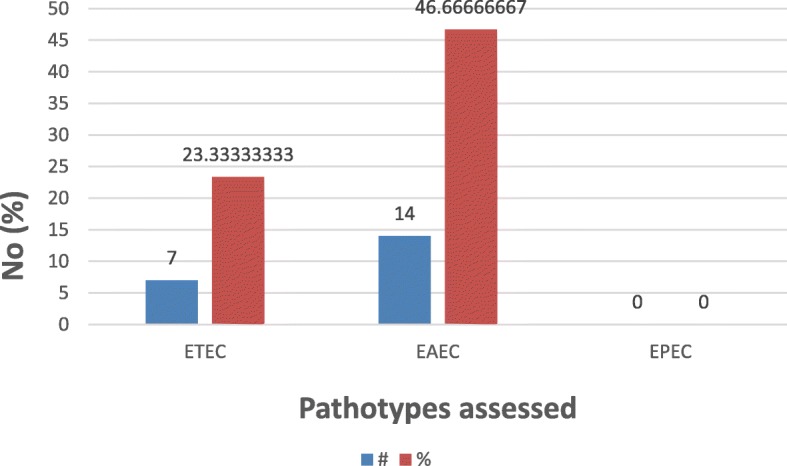
Pathotypes of selected cefotaxime resistant *E. coli* (*n* = 30)

The results of the pathotyping showed that 70% (*n* = 30) of the selected isolates were confirmed as pathogenic; 7 (23.3%) were heat-stable (ST) enterotoxigenic *E. coli* (ETEC), 14 (46.7%) were Enteroaggregative *E. coli* (EAEC), but no enteropathogenic *E. coli* (EPEC) and enteroinvasive *E. coli* (EIEC) were detected. Figure 3 showed the comparison of the various pathotypes.

## Discussion

We conducted this study with the aim of quantifying the cefotaxime-resistant *E. coli* and determining antibiogram to other antibiotics, as well as the presence of beta-lactamase genes in the Gram-negative bacteria from a WWTP from Durban, South Africa. This aim is in line with the critical criteria placed on Enterobacteriaceae resistant to third-generation cephalosporin and carbapenem antibiotics by WHO [14]. The distribution of resistant *E. coli* to the environment through wastewater effluents remains an issue of public health concern, depending on downstream human exposure. A large number of total *E. coli* count (log_10_ CFU/mL) that ranged from 5.7 to 6.8 or cefotaxime-resistant *E. coli* as depicted in Fig. 1(a) - (d) in the influent may not really be of much concern. The former (total *E. coli*) is expected in influent since *E. coli* is a normal part of the gut flora [7, 8]. The presence of the latter (cefotaxime-resistant *E. coli*) might also be due to wastewater from hospital inflow. These resistance attributes are expected from the clinical origin [20, 21, 46] and since the WWTPs are known as early warning system [47], the presence of a large number of cefotaxime-resistant *E. coli* might be a warning sign of probable lack of pretreatment for the hospital outflow.

The effluent concentration of both total *E. coli* and cefotaxime-resistant *E. coli* (range in log_10_ CFU/mL = 0 to 0.3) (Fig. 1e) was low and in most cases zero due to the chlorination practices. Even when the bacterial cells confirmed to be dead after chlorination, studies have shown that large percentage of their genes persists in the effluent [48]. These genes can be picked up by competent environmental strains of *E. coli,* conferring the antibiotic resistance status of the dead organism on the new [27]. These competent cells might accidentally be among other total coliforms with effluent concentration (log_10_ CFU/mL) that ranged from 0 to 3.6. This is possible because transformation only requires a competent cell to pick up resistance genes in the environment, even if the donor is from another genus [27, 49].

Though many cefotaxime-resistant Gram-negative bacteria are already recognized as critical threats [11], their further resistance to other antibiotics within the last line of defense poses a greater threat toward total resistance [50, 51]. Gentamicin resistance (87.5%) detected in our study was in tandem with the reports by Jakobsen et al. [52]. It was revealed that the isolates showed intermediate resistance to meropenem (11.8%) as well as resistance to ciprofloxacin (47.1%) and colistin sulphate (76.5%) by cefotaxime-resistant *E. coli*. Fluoroquinolone resistant isolates of *E. coli* from sewage had also been reported by Colomer-Lluch et al. [53]. The corresponding threat is because fluoroquinolones (e.g. ciprofloxacin) ranked as one of four highest prioritized critically important antimicrobials [54]. A figure of about every 1 in 2 (47.1%) as detected in this study for ciprofloxacin and cefotaxime-resistant *E. coli* is synonymous with having a 12.5% chance of therapeutic failure or total resistance, in line with the position of WHO [50]. The colistin resistance (76.5%) in Enterobacteriae in this study is in worrying tandem with the report of 100% colistin resistance in lake water and 33.3% in sewage found by Fernandes et al. [55]. Resistance to colistin by environmental or clinical isolates is significantly related to resistance to some other antibiotics in the last line of defense [56]. Colistin administration induces an increase in carbapenemase-producing *Enterobacteriaceae* around the world and resistance to colistin itself is directly linked with the agricultural use of human antibiotics [56]. The resistance of 35.3% to amoxicillin-clavulanic acid revealed that the basis of resistance in some of the isolates in this study goes beyond beta-lactamase production. This is because clavulanic acid would have inhibited the effect of the enzyme to ensure susceptibility to the antibiotic. This profile highlighted the bacteria as a potential threat for any less kitted workers in WWTPs due to the risks of occupational exposure [57].

The discharge of the antibiotic-resistant bacteria from WWTPs to the environment is reported widely [32, 49, 51, 52, 58–60]. Concurrent resistance to multiple antibiotics is an issue of greater concern. A wide range of multiple antibiotic resistance indices (0.13–0.75) might be a reflection of wide disparity in the static-use and the adaptive-use of these antibiotics [37]. This may signify a consistent administration of these antibiotics without medical prescription or the organisms originating from hospital environment [60]. Large percentage (> 94%) of the cefotaxime-resistant *E. coli* exhibited multiple antibiotic indices (MAR Index) > 0.2 which means that the isolates might have originated from high risk source (s), where antibiotics are probably abused [38, 40, 59]. As noted earlier, since the WWTPs are known as early warning systems [47], the MAR Indices in this study revealed that antibiotics are in constant abuse and hence, their high selective pressure [61].

The high resistance to colistin sulphate (76.5%) detected in this study is considered alarming. Over the past few years now, attention around the world has been especially focused on the epidemiology of resistance to colistin in bacteria. Researchers link the emergence of colistin resistance to prior exposure to the sublethal concentration by the bacteria, but studies have reported the isolation of colistin-resistant *E. coli* strains from individuals without history of colistin usage [62]. In a study in Laos, Olaitan et al. [63] isolated colistin-resistant *E. coli* from a boy without any record of prior administration of colistin but rather probable zoonotic exposure from the family’s pigs. This is why animal or infected human feces like in waste and wastewater are considerable hotspots [64, 65]. Colistin is used as the final alternative for resistant isolates against β-lactams, aminoglycosides or quinolones, but colistin-resistant Enterobacteriaceae have been classified with pan-drug resistance [66]. So, infections from the *E. coli* isolated from the sampled WWTP can be difficult to treat in an individual if contracted via occupational exposure.

Several antibiotic resistance determinants were detected in this study. These include *bla*_*TEM*_*, bla*_*CTX-M*_*, bla*_*SHV*_*, bla*_*VIM*_*, bla*_*OXA-1*_*, bla*_*KPC-2*_ and *bla*_*NDM-1*_, which might be responsible for some detected resistance to the cefotaxime and other beta-lactam antibiotics [67]. Though *bla*_*VIM*_ was not detected, *bla*_*OXA-1*_*, bla*_*KPC-2*_ and *bla*_*NDM-1*_ might be responsible for intermediate resistance to meropenem by the cefotaxime-resistant *E. coli* [67, 68]. Several beta-lactamase genes like *bla*_*CTX-M*_*, bla*_*SHV*_ and *bla*_*TEM*_ have also been reported in both hospital and municipal wastewater effluents [18, 69]. These reports, as well as our report, indicate the possibility for the determinants to be disseminated among water systems [70], since the genes may persist after the bacterial cells are dead [48]. Concurrent detection of at least two ESBLs’ genes, two carbapenem resistance genes and ESBLs with carbapenem resistance genes (Table 6) depicted the level of threat associated with these bacterial strains.

Reports in other studies [71, 72] showed that ESBL producers from WWTP were *E. coli* and *K. pneumoniae*. Most rampant putative ESBLs’ genes were *bla*_CTX-M_ genes in which the similar gene types were detected in both clinical and environmental isolates [73, 74]. Multi-drug resistant *E. coli* (MDR-*Ec*), have been isolated from a healthy human [75] and the MDR-*Ec* is sometimes shed in feces released into WWTP through the user interface. Intraspecific and interspecific transfer of resistance gene was expected to be high in the WWTP due to high bacterial density. This transfer may include rampant *bla*_CTX-M_ (Coque et al., 2008).

Since 70% (*n* = 30) of the selected isolates were confirmed as pathogenic due to their pathotypes (Fig. 3), their release into the environment may pose threat to public health and to the environment, as these attributes may be widely distributed causing too difficult to control bacterial infection in an epidemic proportion.

## Conclusions

The influent samples in the WWTP contained a large number of Cefotaxime-resistant *E. coli* and other coliforms but the effluent contained only a very scanty concentration of total *E. coli* and other coliforms. Some of the cefotaxime resistant *E. coli* also showed resistance to amoxicillin-clavulanic acid, colistin, meropenem, ciprofloxacin, gentamicin, trimethoprim-sulphamethoxazole, nitrofurantoin and tetracycline. The presence of cefotaxime-resistant and indeed multidrug-resistant Enterobacteriaceae in wastewater influent showed that these organisms, classified with critical threats, were possibly harbored in the population. These, cefotaxime-resistant *E. coli,* were pathogenic as revealed by their pathotypes. These resistant isolates bore extended-spectrum beta-lactamase genes and carbapenem resistance genes singly and concurrently, which make them of potential clinical origin. It is imperative for hospitals to pretreat their wastewater effluent to avoid the critical threat associated with these kinds of bacteria to public health, in case of leakage and human exposure.

## Data Availability

The supporting data are available with the corresponding author and laboratory depositories.

## Abbreviations

CFU: Colony Forming Unit
CLSI: Clinical and Laboratory Standards Institute
EAEC: Enteroaggregative *E. coli*
EPEC: Enteropathogenic *E. coli*
ETEC: Enterotoxigenic *E. coli*
MAR: Multiple Antibiotic Resistance
mL: Milliliter
PCR: Polymerase Chain Reaction
WHO: World Health Organization
WWTP: Wastewater Treatment Plant
μL: Microliter

## References

[1] Edberg SC, Rice EW, Karlin RJ, Allen MJ (2000). *Escherichia coli*: the best biological drinking water indicator for public health protection. Symp Ser Soc Appl Microbiol.

[2] Kaper JB, Nataro JP, Mobley HL (2004). Pathogenic *Escherichia coli*. Nat Rev Microbiol.

[3] Ki V, Rotstein C (2008). Bacterial skin and soft tissue infections in adults: a review of their epidemiology, pathogenesis, diagnosis, treatment and site of care. Can J Infect Dis Med Microbiol.

[4] Allen HK, Donato J, Wang HH, Cloud-Hansen KA, Davies J, Handelsman J (2010). Call of the wild: antibiotic resistance genes in natural environments. Nat Rev Microbiol.

[5] Oteo J, Perez-Vazquez M, Campos J (2010). Extended spectrum [beta]-lactamase producing *Escherichia coli*: changing epidemiology and clinical impact. Curr Opin Infect Dis.

[6] Ferdosi-Shahandashti E, Javanian M, Moradian-Kouchaksaraei M, Yeganeh B, Bijani A, Motevaseli E, Moradian-Kouchaksaraei F (2015). Resistance patterns of *Escherichia coli* causing urinary tract infection. Caspian J Intern Med.

[7] Evans DJ, Evans DG. Escherichia coli in diarrheal disease. In baron S ed. medical microbiology. 4th edition. Galveston (TX); University of Texas Medical Branch at Galveston. 1996; Chapter 25. https://www.ncbi.nlm.nih.gov/books/NBK7710/. 21413261

[8] Anastasi EM, Matthews B, Stratton HM, Katouli M (2012). Pathogenic *Escherichia coli* found in sewage treatment plants and environmental waters. Appl Environ Microbiol.

[9] Tamma PD, Cosgrove SE, Maragakis LL (2012). Combination therapy for treatment of infections with gram-negative Bacteria. Clin Microbiol Rev.

[10] Park SH (2014). Third-generation cephalosporin resistance in gram-negative bacteria in the community: a growing public health concern. Korean J Intern Med.

[11] Section 21 Authorization for Cefotaxime 500 mg Injection, 2019; http://www.kznhealth.gov.za/edlpaed06.pdf.

[12] Koopmans LR, Inlayson HF, Whitelaw A, Decloedt EH, Dramowski A (2018). Paediatric antimicrobial use at a South African hospital. Int J Infect Dis.

[13] Dansey RD, Jacka PJ, Strachan SA, Hay M (1992). Comparison of cefotaxime with ceftriaxone given intramuscularly 12-hourly for community-acquired pneumonia. Diagn Microbiol Infect Dis.

[14] WHO (2017). WHO priority pathogens list for R&D of new antibiotics. 5.

[15] Tian GB, Wang HN, Zou LK, Tang JN, Zhao YW, Ye MY, Tang JY, Zhang Y, Zhang AY, Yang X, Xu CW, Fu YJ (2009). Detection of CTX-M-15, CTX-M-22, and SHV-2 extended- spectrum beta-lactamases ESBLs in *Escherichia coli* fecal-sample isolates from pig farms in China. Foodborne Pathog Dis.

[16] Mir RA, Weppelmann TA, Johnson JA, Archer D, Morris JG, Jeong KC (2016). Identification and Characterization of Cefotaxime Resistant Bacteria in Beef Cattle. PLoS ONE.

[17] Amaya E, Reyes D, Paniagua M, Calderon S, Rashid MU, Colque P, Kuhn I, Mollby R (2012). Antibiotic resistance patterns of Escherichia coli isolates from different aquatic environmental sources in Leon, Nicaragua. Clin Microbiol Infect.

[18] Korzeniewska E, Harnisz M (2013). Beta-lactamase producing Enterobacteriaceae in hospital effluents. J Environ Man.

[19] D’Andrea MM, Arena F, Pallecchi L, Rossolini GM (2013). CTX-M-type beta-lactamases: a successful story of antibiotic resistance. Int J Med Microbiol.

[20] Bora A, Hazarika NK, Shukla SK, Prasad KN, Sarma JB, Ahmed G (2014). Prevalence of *bla*_*TEM*_*, bla*_*SHV*_ and *bla*_*CTX-M*_ genes in clinical isolates of *Escherichia coli* and *Klebsiella pneumoniae* from Northeast India. Indian J Pathol Microbiol.

[21] Ojdana D, Sacha P, Wieczorek P, Czaban S, Michalska A, Jaworowska J, Jurczak A, Poniatowski B, Tryniszewska E (2014). The occurrence of *bla*_*CTX-M*_, *bla*_*SHV*_, and *bla*_*TEM*_ genes in extended-Spectrum β-lactamase-positive strains of *Klebsiella pneumoniae*, *Escherichia coli*, and *Proteus mirabilis* in Poland. Int J Antibiotics.

[22] Diwan V, Chandran SP, Tamhankar AJ, Stålsby LC, Macaden RJ (2012). Identification of extended-spectrum β-lactamase and quinolone resistance genes in *Escherichia coli* isolated from hospital wastewater from Central India. Antimicrob Chemother.

[23] Zhao C, Xie W, Zhang W, Ye Z, Wu H (2014). Mechanism of drug resistance of carbapenems- resistant *Acinetobacter baumannii* and the application of a combination of drugs in vitro. Chin J Burns.

[24] Kumarasamy KK, Toleman MA, Walsh TR, Bagaria J, Butt F, Balakrishnan R (2010). Emergence of a new antibiotic resistance mechanism in India, Pakistan, and the UK: a molecular, biological, and epidemiological study. Lancet Infect Dis.

[25] Pitout JD (2012). Extraintestinal pathogenic *Escherichia coli*: a combination of virulence with antibiotic resistance. Front Microbiol.

[26] Basode VK, Abdulhaq A, Alamoudi MUA, Tohari HM, Quhal WA, Madkhali AM, Hobani YH, Hershan AA (2018). Prevalence of a carbapenem-resistance gene (KPC), vancomycin- resistance genes (van a/B) and a methicillin-resistance gene (mecA) in hospital and municipal sewage in a southwestern province of Saudi Arabia. BMC Res Notes.

[27] Von-Wintersdorff CJH, Penders J, Van Niekerk JM, Mills ND, Majumder S, Van Alphen LB, Savelkoul PHM, Wolffs PFG (2016). Dissemination of antimicrobial resistance in microbial ecosystems through horizontal gene transfer. Front Microbiol.

[28] Vaidya VK (2011). Horizontal transfer of antimicrobial resistance by extended-spectrum β lactamase-producing enterobacteriaceae. J Lab Physicians.

[29] Yin Q, Yue D, Peng Y, Liu Y, Xiao L (2013). Occurrence and distribution of antibiotic- resistant bacteria and transfer of resistance genes in Lake Taihu. Microbes Environ.

[30] Taylor NG, Verner-Jeffreys DW, Baker-Austin C (2011). Aquatic systems: maintaining, mixing and mobilising antimicrobial resistance?. Trends Ecol Evol.

[31] Baquero F, Martinez JL, Canton R (2008). Antibiotics and antibiotic resistance in water environments. Curr Opin Biotechnol.

[32] Dolejska M, Frolkova P, Florek M, Jamborova I, Purgertova M, Kutilova I, Cizek A, Guenther S, Literak I (2011). CTXM-15-producing *Escherichia coli* clone B2-O25b-ST131 and *Klebsiella* spp. isolates in municipal wastewater treatment plant effluents. J Antimicrob Chemother.

[33] Salehi TZ, Madani SA, Karimi V, Khazaeli FA (2008). Molecular genetic differentiation of avian *Escherichia coli* by RAPD-PCR. Braz J Microbiol.

[34] Yokoigawa K, Takikawa A, Kawai H (1999). Difference between *Escherichia coli* O157:H7 and non-pathogenic *E. coli*: survival and growth in seasonings. J Biosci Bioeng.

[35] Daly P, Collier T, Doyle S (2002). PCR-ELISA detection of *Escherichia coli* in milk. Lett Appl Microbiol.

[36] Cheesebrough M. District Laboratory Practice in Tropical Countries. Part 2. 2nd ed. Cambridge: Cambridge University Press; 2006. p. 132–43.

[37] CLSI, Clinical and Laboratory Standards Institute (2017). Performance standards for antimicrobial susceptibility testing.

[38] Krumperman PH (1983). Multiple antibiotic resistance indexing of *E. coli* to identify high-risk sources of fecal contamination of foods. Appl Environ Microbiol.

[39] Samuel L, Marian MM, Apun K, Lesley MB, Son R (2011). Characterization of *E. coli* isolated from cultured catfish by antibiotic resistance and RAPD analysis. Int Food Res J.

[40] Adegoke AA, Okoh AI (2014). Species diversity and antibiotic resistance properties of Staphylococcus of farm animal origin in Nkonkobe Municipality, South Africa. Folia Microbiol (Praha).

[41] Yang F, Mao D, Zhou H, Luo Y (2016). Prevalence and Fate of Carbapenemase Genes in a Wastewater Treatment Plant in Northern China. PLoS ONE.

[42] Khurana S, Mathur P, Kapil A, Valsan C, Behera B (2017). Molecular epidemiology of beta- lactamase producing nosocomial gram-negative pathogens from north and south Indian hospitals. J Med Microbiol.

[43] Omar KB. Determining the pathogenicity and quantities of *Escherichia coli* in selected south African water types using molecular biology techniques: M.Tech Biotechnology Thesis. Johannesburg: University of Johannesburg; 2007.

[44] Vilchez S, Reyes D, Paniagua M, Bucardo F, Mollby R, Weintraub A (2009). Prevalence of diarrhoeagenic *Escherichia coli* in children from Leon, Nicaragua. J Med Microbiol.

[45] Matar GM, Adbo D, Khneisser I, Youssef M, Zouheiry H, Adbelnour G, Harakeh HS (2002). The multiplex-PCR based detection and genotyping of diarrhoeagenic *Escherichia coli* in diarrhoea stools. Ann Trop Med Parasitol.

[46] Lien LTQ, Lan PT, Chuc NTK, Hoa NQ, Nhung PH, Thoa NTM, Diwan V, Tamhankar AJ, Lundborg CS (2017). Antibiotic resistance and antibiotic resistance genes in *Escherichia coli* isolates from hospital wastewater in Vietnam. Int J Environ Res Public Health.

[47] Jurga A, Gemza N, Janiak K (2017). A concept development of an early warning system for toxic sewage detection. E3S Web Conferences.

[48] Lood R, Ertürk G, Mattiasson B (2017). Revisiting antibiotic resistance spreading in wastewater treatment plants – bacteriophages as a much-neglected potential transmission vehicle. Front Microbiol.

[49] Chen I, Dubnau D (2004). DNA uptake during bacterial transformation. Nat Rev Microbiol.

[50] Poirel L, Nordmann P (2006). Carbapenem resistance in *Acinetobacter baumannii*: mechanisms and epidemiology. Clin Microbiol Infect.

[51] Fischer J, Rodríguez I, Schmoger S, Friese A, Roesler U, Helmuth R, Guerra B (2012). *Escherichia coli* producing VIM-1 carbapenemase isolated on a pig farm. J Antimicrob Chemother.

[52] Jakobsen L, Sandvang D, Hansen LH, Bagger-Skjot L, Westh H, Jorgensen C, Hansen DS, Pedersen BM, Monnet DL, Frimodt-Moller N, Sorensen SJ, Hammerum AM (2008). Characterisation, dissemination and persistence of gentamicin resistant *Escherichia coli* from a Danish university hospital to the waste water environment. Environ Int.

[53] Colomer-Lluch M, Mora A, López C, Mamani R, Dahbi G, Marzoa J, Herrera A, Viso S, Blanco JE, Blanco M, Alonso MP, Jofre J, Muniesa M, Blanco J (2013). Detection of quinolone-resistant *Escherichia coli* isolates belonging to clonal groups O25b:H4-B2-ST131 and O25b:H4-D-ST69 in raw sewage and river water in Barcelona, Spain. J Antimicrob Chemother.

[54] World Health Organisation Advisory Group on Integrated Surveillance of Antimicrobial Resistance (2012). Critically Important Antimicrobials for Human Medicine-3rd Revision.

[55] Fernandes MR, Moura Q, Sartori L, Silva KC, Cunha MP, Esposito F, Lopes R, Otutumi LK, Gonçalves DD, Dropa M, Matté MH, Monte DF, Landgraf M, Francisco GR, Bueno MF, de- Oliveira Garcia D, Knöbl T, Moreno AM, Lincopan N. Silent dissemination of colistin-resistant *Escherichia coli* in South America could contribute to the global spread of the mcr-1 gene. Euro Surveill. 2016;21(17):1–6. 10.2807/1560-7917.ES.2016.21.17.30214 27168587

[56] Hao H, Cheng G, Iqbal Z, Ai X, Hussain HI, Huang L (2014). Benefits and risks of antimicrobial use in food-producing animals. Front Microbiol.

[57] Leonard AFC, Zhang L, Balfour AJ, Garside R, Hawkey PM, Murray AK, Ukoumunne OC, Gaze WH. Exposure to and colonisation by antibiotic-resistant *E. coli* in UK coastal water users: Environmental surveillance, exposure assessment, and epidemiological study (Beach Bum Survey). Environ Int. 2018;114:326–33. 10.1016/j.envint.2017.11.003 29343413

[58] Chagas TP, Seki LM, Cury JC, Oliveira JA, Dávila AM, Silva DM, Asensi MD (2011). Multiresistance, beta-lactamase encoding genes and bacterial diversity in hospital wastewater in Rio de Janeiro, Brazil. J Appl Microbiol.

[59] Sabate M, Prats G, Moreno E, Balleste E, Blanch AR, Andreu A (2008). Virulence and antimicrobial resistance profiles among *Escherichia coli* strains isolated from human and animal wastewater. Res Microbiol.

[60] Laxminarayan R, Klugman KP (2011). Communicating trends in resistance using a drug resistance index. BMJ Open.

[61] Suresh T, Srinivasan D, Hatha AAM, Lakshmanaperumalsamy P (2000). The incidence, antibiotics resistance, and survival of Salmonella and Escherichia coli isolated from broiler chicken retail outlets. Microb Environ.

[62] Olaitan AO, Morand S, Rolain JM (2016). Emergence of colistin-resistant bacteria in humans without colistin usage: a new worry and cause for vigilance. Int J Antimicrob Agents.

[63] Olaitan AO, Thongmalayvong B, Akkhavong K, Somphavong S, Paboriboune P, Khounsy S, Rolain JM (2015). Clonal transmission of a colistin-resistant *Escherichia coli* from a domesticated pig to a human in Laos. J Antimicrob Chemother.

[64] Newton RJ, McLellan SL, Dila DK, Vineis JH, Morrison HG, Eren AM, Sogin ML (2015). Sewage reflects the microbiomes of human populations. MBio..

[65] Drieux L, Haenn S, Moulin L, Jarlier V (2016). Quantitative evaluation of extended-spectrum beta- lactamase-producing *Escherichia coli* strains in the wastewater of a French teaching hospital and relation to patient strain. Antimicrob Resist Infect Control.

[66] Biswas S, Brunel JM, Dubus JC, Reynaud-Gaubert M, Rolain JM (2012). Colistin: an update on the antibiotic of the 21st century. Expert Rev Anti-Infect Ther.

[67] Kooti S, Motamedifar M, Sarvari J (2000). Antibiotic Resistance Profile and Distribution of Oxacillinase Genes Among Clinical Isolates of *Acinetobacter baumannii* in Shiraz Teaching Hospitals, 2012–2013. Jundishapur J Microbiol.

[68] Vijayakumar S, Gopi R, Gunasekaran P, Kamini MB, Shalini W, Veeraraghavan AB (2016). Molecular characterization of invasive Carbapenem-resistant *Acinetobacter baumannii* from a tertiary Care Hospital in South India. Infect Dis Ther.

[69] Korzeniewska E, Korzeniewska A, Harnisz M (2013). Antibiotic resistant *Escherichia coli* in hospital and municipal sewage and their emission to the environment. Ecotoxicol Environ Saf.

[70] Tacão M, Correia A, Henriques I (2012). Resistance to broad-spectrum antibiotics in aquatic systems: anthropogenic activities modulate the dissemination of Bla(CTX-M)-like genes. Appl Environ Microbiol.

[71] Conte D, Palmeiro JK, da Silva NK, de Lima TM, Cardoso MA, Pontarolo R, Degaut Pontes FL, Dalla-Costa LM (2017). Characterization of CTX-M enzymes, quinolone resistance determinants, and antimicrobial residues from hospital sewage, wastewater treatment plant, and river water. Ecotoxicol Environ Saf.

[72] Dolejska M, Frolkova P, Florek M, Jamborova I, Purgertova M, Kutilova I, Cizek A, Guenther S, Literak I (2011). CTX-M-15-producing *Escherichia coli* clone B2-O25bST131 and *Klebsiella* spp. isolates in municipal wastewater treatment plant effluents. J Antimicrob Chemother.

[73] Tacão M, Correia A, Henriques I (2012). Resistance to broad-spectrum antibiotics in aquatic systems, anthropogenic activities modulate the dissemination of bla, CTXM-like genes. Appl Environ Microbiol.

[74] Rodrigues C, Machado E, Fernandes S, Peixe L, Novais Â (2016). An update on faecal carriage of ESBL producing Enterobacteriaceae by Portuguese healthy humans, detection of the H30 subclone of B2-ST131 *Escherichia coli* producing CTX-M-27. J Antimicrob Chemother.

[75] Jakobsen L, Garneau P, Kurbasic A, Bruant G, Stegger M, Harel J, Jensen KS, Brousseau R, Hammerum AM, Frimodt-Moller N. Microarray-based detection of extended virulence and antimicrobial resistance gene profiles in phylogroup B2 *Escherichia coli* of human, meat and animal origin. J Med Microbiol. 2011;60(10):1502–11. 10.1099/jmm.0.033993-0 21617024

